# Crosstalk of Redox-Related Subtypes, Establishment of a Prognostic Model and Immune Responses in Endometrial Carcinoma

**DOI:** 10.3390/cancers14143383

**Published:** 2022-07-12

**Authors:** Rui Geng, Jiahang Song, Zihang Zhong, Senmiao Ni, Wen Liu, Zhiqiang He, Shilin Gan, Qinghao Huang, Hao Yu, Jianling Bai, Jinhui Liu

**Affiliations:** 1 Department of Biostatistics, School of Public Heath, Nanjing Medical University, Nanjing 210029, China; gengrui@njmu.edu.cn (R.G.); zhzhong@njmu.edu.cn (Z.Z.); senmiaoni@njmu.edu.cn (S.N.); liuwen@njmu.edu.cn (W.L.); zqianghe@163.com (Z.H.); ganshilin@njmu.edu.cn (S.G.); qinghaohuang@njmu.edu.cn (Q.H.); haoyu@njmu.edu.cn (H.Y.); 2 Department of Radiation Oncology, The First Affiliated Hospital of Nanjing Medical University, Nanjing 210029, China; songjiahang001@163.com; 3 Department of Gynecology, The First Affiliated Hospital of Nanjing Medical University, Nanjing 210029, China

**Keywords:** redox, endometrial carcinoma, immune infiltration, drug sensitivity

## Abstract

**Simple Summary:**

In order to explore the role of redox as a prognostic indicator in endometrial carcinoma (EC), we detected the expression patterns of 55 redox-related genes (RRGs) in EC cohorts from public databases. Performing consensus cluster algorithm, we determined four molecular subclusters based on RRGs which had significant differences in overall survival (OS) and immune activities of EC patients. Furthermore, we developed a prognostic risk model on the basis of the redox-related subtype by stepwise Cox regression analyses. All EC patients were divided into high-risk and low-risk groups according to the median value of risk score. Our proposed model could accurately assess the clinical outcome and had favorable independent ability in EC cases. Moreover, our signature can serve as a predictor for immune status and chemotherapy sensitivity.

**Abstract:**

Redox plays a central part in the pathogeneses and development of tumors. We comprehensively determined the expression patterns of redox-related genes (RRGs) in endometrial carcinoma (EC) cohorts from public databases and identified four different RRG-related clusters. The prognosis and the characteristics of TME cell infiltration of RRGcluster C patients were worse than those of other RRG clusters. When it comes to the gene cluster, there were great differences in clinicopathology traits and immunocyte infiltration. The RRG score was calculated by Cox analyses, and an RRG-based signature was developed. The risk score performed well in the EC cohort. Samples were separated into two risk subgroups with the standard of the value of the median risk score. Low-risk patients had a better prognosis and higher immunogenicity. In addition, RRG score was closely associated with immunophenoscore, microsatellite instability, tumor mutation burden, tumor stem cell index, copy number variation and chemotherapy sensitivity. The nomogram accurately predicted the prognosis of patients, and our model showed better performance than other published models. In conclusion, we built a prognostic model of RRGs which can help to evaluate clinical outcomes and guide more effective treatment.

## 1. Introduction

Endometrial carcinoma (EC) is a common cancer in female reproductive organs in developed countries. With the increase of aging and obesity in the population, the incidence rate and mortality rate of EC continue to rise [1,2,3]. It is believed that there will be 65,950 new cases and 12,550 deaths in the United States in 2022 [4]. Some patients were diagnosed in the advanced stage or occult metastasis stage [5] and were prone to tumor recurrence, which always had a poor prognosis [6]. Due to the high heterogeneity of EC patients [1,7], the traditional clinical factors-based prognosis systems have limited significance for prognosis judgment and treatment guidance of patients [5,8]. Researchers are attempting to identify new biomarkers, improving the risk prediction system by combining molecular characteristics and traditional clinicopathological factors [7]. 

Tumor cells carrying characteristic genomic changes exist in the microenvironment of their host. Tumor cells, stromal cells and immune cells constitute the tumor microenvironment (TME), which promotes tumor malignant transformation to a large extent [9]. The interactions between tumor cells and the TME is the decisive parameter of oncogenesis, progression, therapeutic drug resistance and clinical outcome. The TME can promote tumor cell proliferation and subsequent metastasis-related phenotypes. Moreover, it can be actively involved in the study of drug-induced drug resistance in cancer cells [10]. 

Immunotherapy has been presented as an alternative or supplementary remedy for cancer. Great efficacy has been shown when PD-1 and PD-L1 antibodies are used in cancer treatment [11]. Cancer cells upregulate negative immune checkpoints through tumor-infiltrating immunocytes, leading to host immune surveillance and cancer progression [5].

Redox is tightly bound up with the activation of the TME and tumor survival [12]. Compared with traditional targets, the redox species of the TME are known as effective “regulators” and “targets” of antitumor therapy [12,13]. In addition, the imbalance of redox homeostasis can lead to cancer cell apoptosis [14]. Apoptosis has been proved to have the capacity to inhibit the proliferation of EC cells [15]. The curative effect of several drugs related to redox have achieved encouraging results in preclinical and clinical studies. For example, thioredoxin and thioredoxin reductase are crucial constituent parts of the thioredoxin system and are considered to be vital regulators of tumor development [16]. Therefore, the prognostic model customized for redox offers a promising new strategy for the diagnosis and treatment of EC [17].

## 2. Materials and Methods

### 2.1. Data Sources

Gene expression profiles of EC and the related clinicopathological information were collected from The Cancer Genome Atlas (TCGA) (https://portal.gdc.cancer.gov/)(accessed on 13 January 2022) and Gene Expression Omnibus (GEO) (https://www.ncbi.nlm.nih.gov/geo/)(accessed on 13 January 2022). Cases without complete clinical data were eliminated to reduce statistical bias. Finally, 562 samples from TCGA-UCEC and GSE119041 were utilized for subsequent study. The details of these patients are shown in Table S1. Fragments per kilobase values of TCGA-UCEC were changed to transcripts per kilobase million for differential analysis [18]. Normalization and removal of batch effects between the TCGA-UCEC and GEO datasets were performed through the “ComBat” algorithm [19].

### 2.2. Consensus Clustering Analysis of RRGs

Two redox-related gene (RRG) sets were collected from MSigDB (https://www.gsea-msigdb.org/gsea/msigdb) (accessed on 13 January 2022) and a total of 55 RRGs were collected. Consistency cluster analysis was employed by the “ConsensusClusterPlus” package in R and the patients were divided into different subtypes in light of the expression of the RRGs mentioned above [20]. This process was repeated 1000 times to assure the stability of typing. In addition, principal component analysis (PCA) was implemented by the “ggplot2” R package.

### 2.3. Functional Annotation and Enrichment Analysis

Differentially expressed genes (DEGs) between the clusters were identified by the R package “limma” with the standard of adjusted *p* value < 0.05 [21]. Next, two different gene clusters were identified by the consistent clustering algorithm. Gene Ontology and Kyoto Encyclopedia of Genes and Genomes analyses were applied to detect the underlying function of DEGs by the R package “clusterprofiler” [22,23].

### 2.4. Establishment of the Prognostic Model in Light of RRGs

The TCGA-UCEC cohort was used as training set, while samples from GSE119041 and the set consisting of the TCGA-UCEC cohort and GSE119041 were utilized to confirm the prediction capacity of the model. In the training group, univariate Cox analysis was applied to determine the DEGs associated with the survival of EC patients. Then, least absolute shrinkage and selection operator (LASSO) regression was employed to minimize the risk of over fitting by the “glmnet” package [24]. Finally, multivariate Cox analysis was applied to screen candidate RRGs to establish a prognostic RRG-based signature (RBS). The formula was as follows: $risk\;score=\sum_{i=1}^{n}\left(Coef_{i}\times {\mathrm{RRGexp}}_{i}\right)$, where coef and exp mean the coefficient and expression level of each gene, respectively. According to the median risk score, patients were separated to two risk groups. The “survminer” package was employed to perform Kaplan–Meier survival analysis. 

### 2.5. Stratification Analyses 

Chi-square tests were employed to analyze the correlations between RRG score and clinicopathological features (age, grade, fustat, stage, histological type). To estimate the independence of the RBS, we utilized univariate and multivariate Cox analysis on the set consisting of TCGA-UCEC and GSE119041. In addition, we also conducted stratified analysis according to age, grade, stage and historical type to further examine the predictive ability of the model.

### 2.6. Correlation between the RBS and Other Biological Processes

Rosenberg et al. identified biological process-associated gene sets, including epithelial mesenchymal transition markers, DNA damage repair, nucleotide excision repair and the CD8 T-effector signature [25]. We performed a correlation analysis of the immune infiltration score of these biological pathways between the two subtypes.

### 2.7. Exploration of Immune Status between Different Subgroups

The ESTIMATE algorithm was employed to predict the immune status through the R package “estimate” [26]. Single sample gene set enrichment analysis (ssGSEA) was employed to evaluate the distinction of immunocyte and immunity between different subgroups [27]. It can quantitatively evaluate the composition of immune cells from gene expression data [28]. To calculate the fraction of tumor-infiltrating immune cells (TIICs), CIBERSORTx was employed (https://cibersortx.stanford.edu/) (accessed on 13 January 2022). We analyzed the association between 22 TIIC scores and risk scores. A bubble plot was drawn to display the positive and negative correlation between RRG score and immune cell types. 

### 2.8. Prediction of Immunotherapy Response

Evaluation of immunophenoscore (IPS) based on gene expression Z-score was computed in accordance with gene expression levels in representative cells, ranging from 0 to 10 [29]. Tumor mutation burden (TMB) means the number of mutations per megabase of DNA sequenced in a particular cancer [30]. It can be employed to reflect the response of checkpoint blocking immunotherapy [31]. After analyzing from The Cancer Immunome Atlas database (https://www.tcia.at/home) (accessed on 13 January 2022), we obtained the MSI data, which is a powerful factor for evaluating the clinical outcome of patients [32]. 

### 2.9. Phenotypes of DNAss and RNAss Differentiation

A cancer stem cell score was designed to gauge cancer stem cell association [33]. The score ranges from 0 to 1. The closer the score approaches to 1, the stronger the degree of stemness and the lower the degree of differentiation. Both RNAss and DNAss scores were collected from the Xena browser (https://xenabrowser.net/datapages/) (accessed on 13 January 2022).

### 2.10. Assessment of Drug Sensitivity

In order to estimate the efficacy of chemotherapeutic drugs on EC patients, the half maximum inhibitor concentration (IC50) of chemotherapeutic drugs was generated by the “pRRophetic” R package. We obtained and calculated drug sensitivity information according to the CellMiner database (http://discover.nci.nih.gov/cellminer/) (accessed on 13 January 2022) [34]. 

### 2.11. Construction of a Nomograph System

The R package “rms” was employed to construct a nomograph system visualizing the role of various factors in predicting the prognosis of EC according to clinical characteristics and risk score [35]. The restricted mean survival (RMS) package was employed to assess the C-index for all features. Decision curve analysis assesses the clinical application of the RBS by evaluating the net benefit rate. The prediction performance of the RBS was achieved through c-index and area under the curve (AUC) [36,37]. 

### 2.12. Statistical Analysis

*p* < 0.05 was considered as statistically significance. All data were input into R4.1.0 software for processing. The distinction between the two groups were evaluated by Student’s *t*-test and analysis of variance. Spearman and distance correlation analyses were applied to estimate relationship coefficients between the expression of RRGs and infiltrating immune cells.

## 3. Results

### 3.1. Genetic Features of RRGs in EC

The research process of our study is displayed in Figure S1.

**Figure 1:**
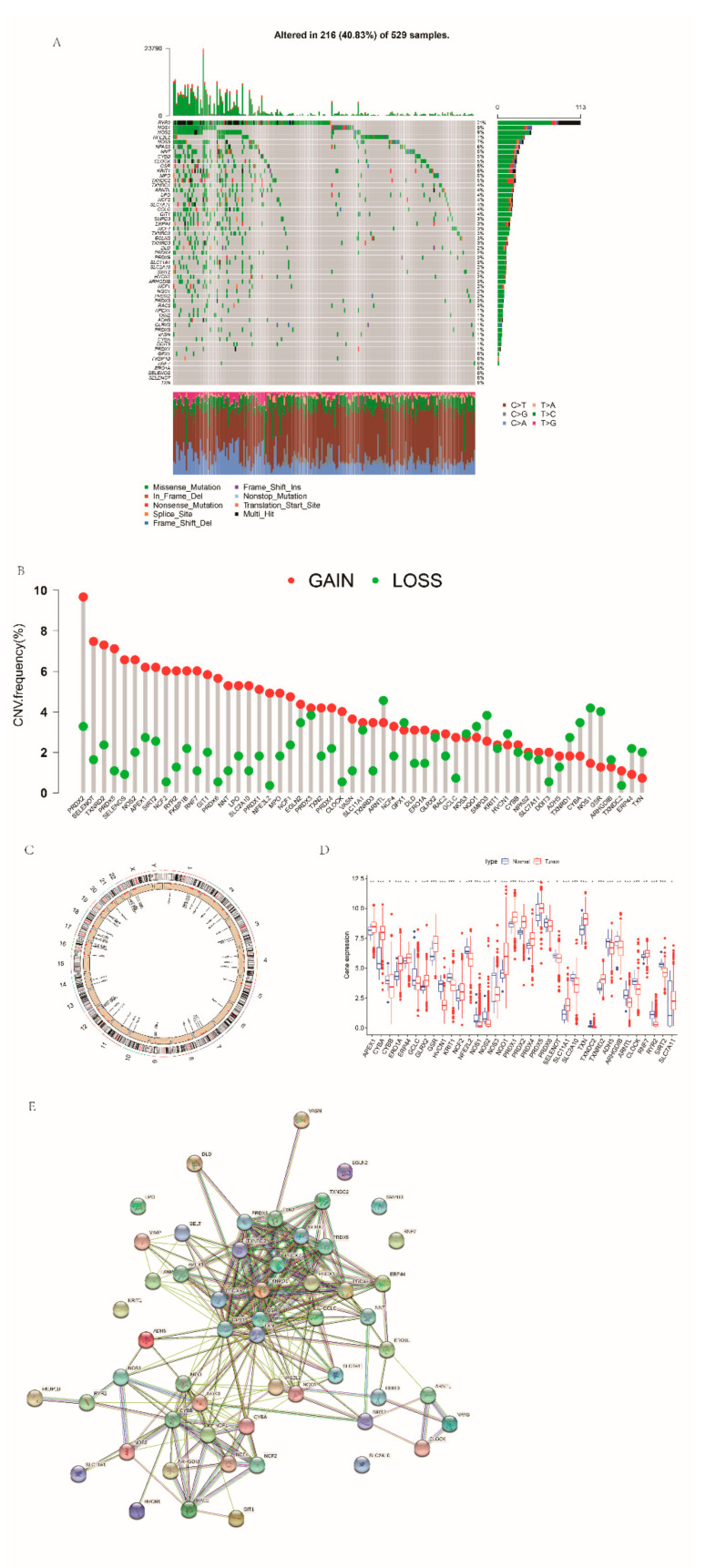
Genetic features of RRGs in EC: (**A**) mutations occurring in 216 of 529 EC patients; (**B**) CNV frequency in RRGs; (**C**) the location of CNV changes on 23 chromosomes in RRGs; (**D**) the differences of RRG expression levels between normal and EC tissues; and (**E**) the PPI network of RRGs.

Firstly, we carried out a summary analysis of the somatic mutation status of these 55 RRGs (Figure 1A). Among 529 samples, 216 samples were mutated, and the incidence of somatic mutation was 40.83%. The mutation frequency of RYR2 was the highest, which was 21%, followed by NOS1 and NOS2. GPX1, FKBP1B, RNF7, ERO1A, SELENOS, SELENOT and TXN had no mutation. Then, we analyzed the copy number variations (CNVs) of these genes. CNVs occurred in almost all genes, with the greatest gain in PRDX2 (Figure 1B). The RRGs’ chromosomal locations of the CNV alterations are revealed in Figure 1C. The expression levels of most of these RRGs between normal and tumor tissues were different (Figure 1D). The protein–protein interaction network diagram shows the interrelationships that exist between these genes (Figure 1E). In addition, we screened out 34 RRGs presenting significant prognostic values (Figure S2).

### 3.2. Identification of Redox-Associated Molecular Subtype in EC

**Figure 2:**
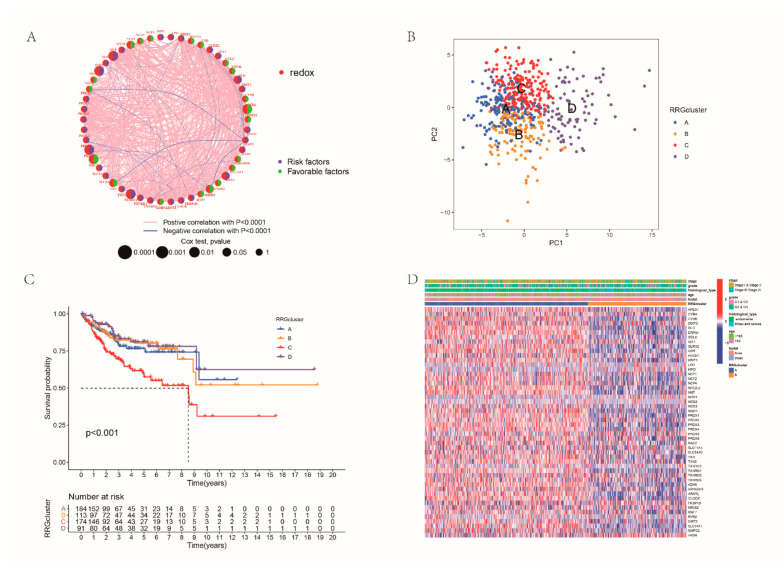
Consistent clustering analysis: (**A**) interactions among RRGs in EC; (**B**) the result of PCA analysis; (**C**) difference in survival probability between RRG clusters; and (**D**) differences in clinicopathologic features between the two RRG subtypes.

We first integrated samples from the TCGA-UCEC and GSE119041 cohort. Figure 2A reveals the interactions and prognostic value of all RRGs (Figure 2A). To determine a novel molecular subtype in EC, the consistent cluster method was conducted according to the expression profiles of RRGs, and all patients were divided into four RRG clusters (Figure S3). PCA showed significant differences in redox transcriptional profiles between four RRG clusters (Figure 2B). Survival analysis displayed that the people in cluster C had the shortest OS (Figure 2C). Furthermore, significant differences in clinicopathological characteristics were shown between the four clusters (Figure 2D). RRGcluster A was preferentially related to younger age, earlier stage and grade, lighter histological types and better survival status. 

### 3.3. Characteristics of the TME in Distinct RRG Clusters

**Figure 3:**
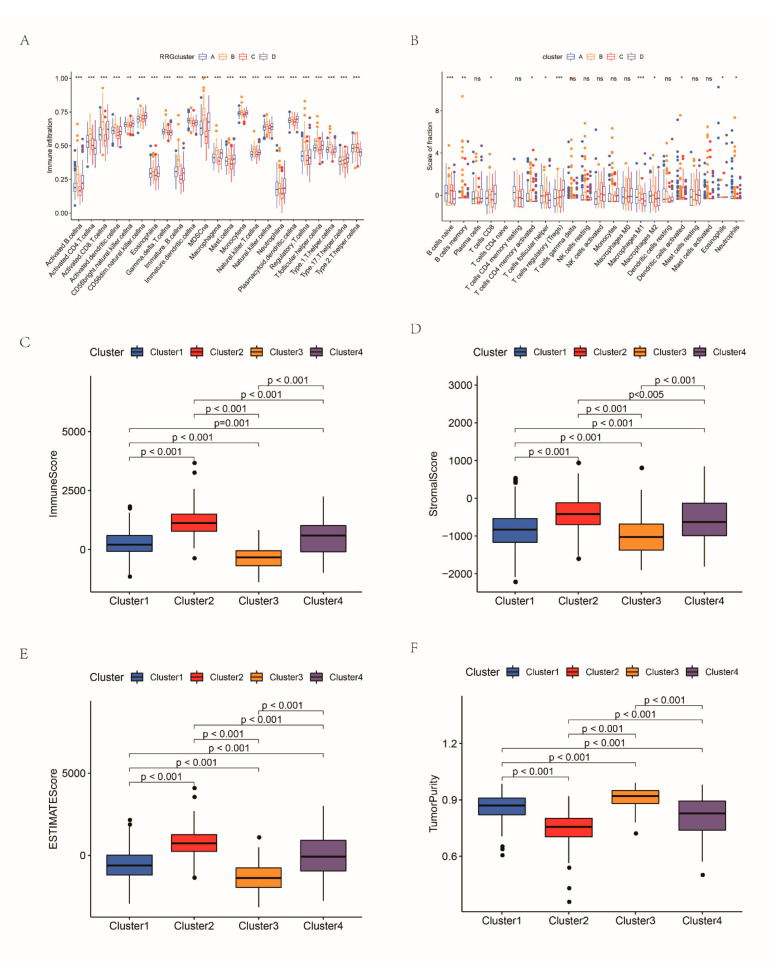

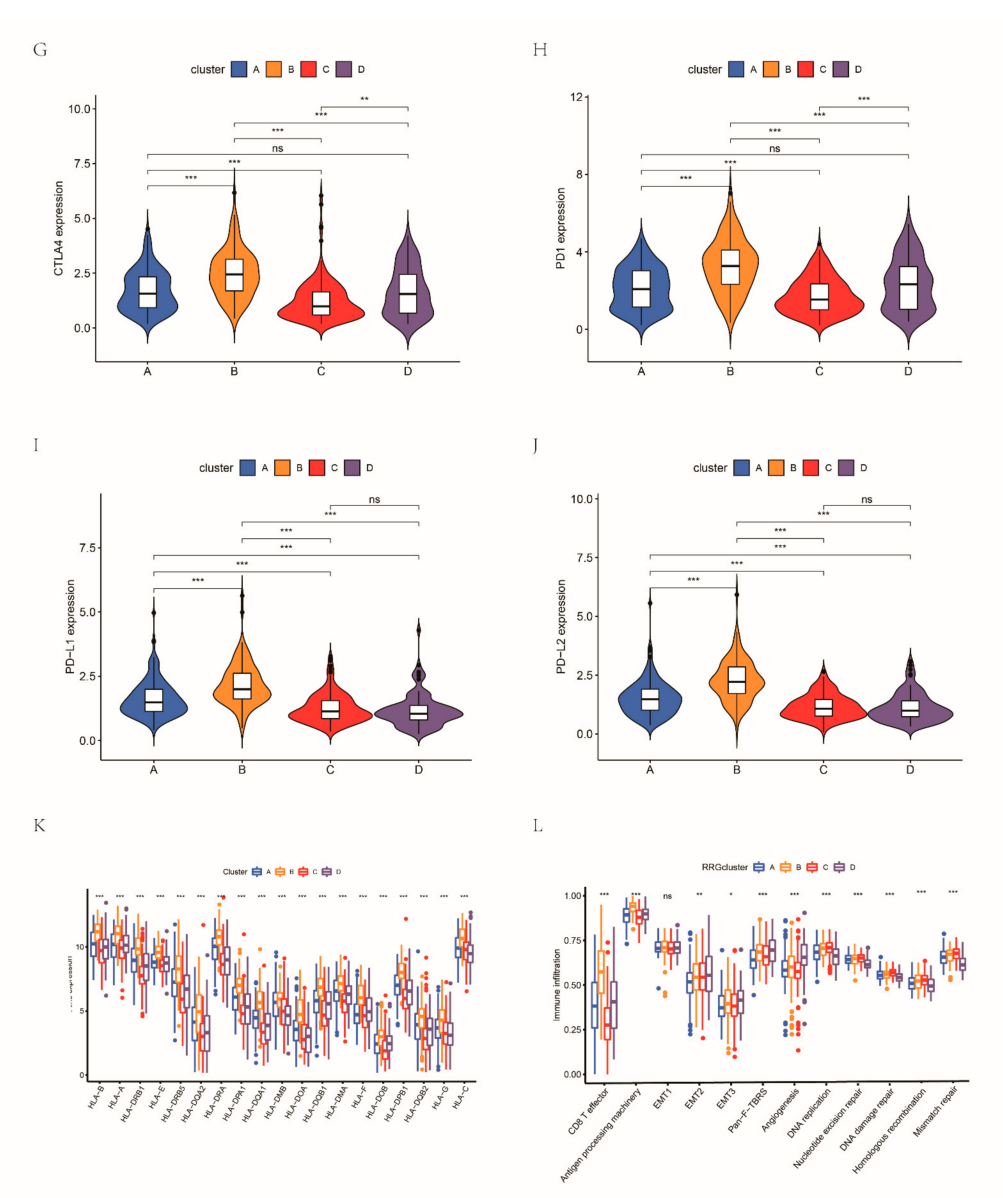
The TME of the four RRG clusters: (**A**) comparison of immune infiltration levels of the RRG clusters; (**B**) the differences of 22 TIICs between the four RRG clusters; comparison of the stromal score (**C**), immune score (**D**), estimated score (**E**) and tumor purity (**F**) of the RRG clusters; (**G**–**J**) expression levels of immune checkpoints between four RRG clusters; (**K**) HLA expression levels of RRG clusters; and (**L**) comparison of the scores of biological processes between the RRG clusters. * *p* < 0.05; ** *p* < 0.01; *** *p* < 0.001; ns, not significant.

For the purpose of deeply analyzing the potential biological significance of these four clusters, GSVA was conducted. The enrichment of cluster D was significantly different from that of the other three clusters; we found that the enrichment levels of some processes related to immune activation in cluster D were the lowest, suggesting cluster D groups tend to develop immunosuppression (Figures S4 and S5). Then, ssGSEA analysis was conducted to evaluate immune infiltration in these clusters. We noticed that the immune infiltration of cluster B was very abundant, and the degree of infiltration was significantly higher than that of the other RRG clusters. Innate and adaptive immune cells, such as B cells, CD4 T cells, CD8 T cells, macrophages and natural killer cells, were significantly enriched in RRGcluster B (Figure 3A). Then, we evaluated the differences of 22 TIICs between the four RRG clusters (Figure 3B). Naïve B cells, regulatory T cells (Tregs) and macrophages had the most significant difference among the four RRG clusters. In addition, we applied ESTIMATE to generate three types of TME scores in the RRG clusters and evaluated the tumor purity, which indicated that the TME score of RRGcluster B was the highest and that of RRGcluster C was the lowest (Figure 3C–F). The tumor purity was the opposite. Based on these analyses, we noted that the four RRG clusters had different immune infiltration. Among them, RRGcluster C corresponds to “cold” tumors, which are characterized by less invasive immune cells and a weak response to immunotherapy, while RRGcluster B is roughly equivalent to “hot” tumors, which are characterized by more active immune cell infiltration and will receive more benefits from immunotherapy. Comparing the expression levels of immune checkpoints, it was found that CTLA4, PD1, PD-L1 and PD-L2 (Figure 3G–J) were highly expressed in RRGcluster B. The human leukocyte antigen (HLA) expression levels of RRG clusters were also significantly different (Figure 3L). Figure 3 suggests that some immune biological processes were more prominent in RRGcluster B, including CD8 T effector and antigen processing machine (Figure 3).

### 3.4. Identification of Gene Clusters Based on DEGs

**Figure 4:**
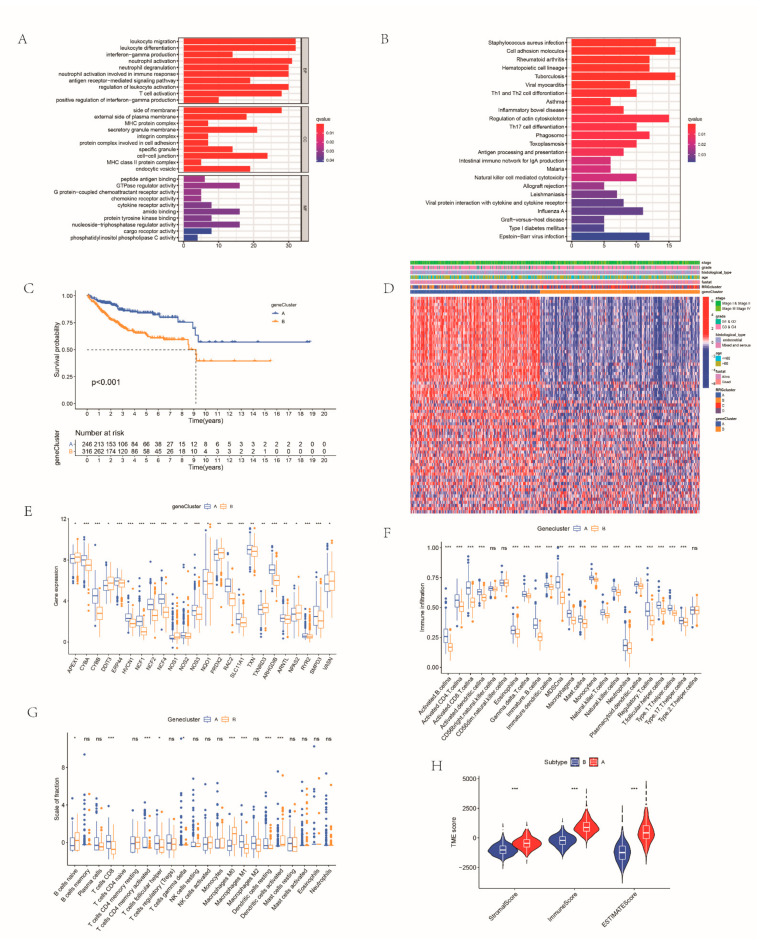
Gene subtype identification based on DEGs: Gene Ontology (**A**) and Kyoto Encyclopedia of Genes and Genomes (**B**) analysis of the four RRG clusters; (**C**) survival probability of the gene subtypes; (**D**) associations between clinicopathologic characteristics and the gene clusters; (**E**) the expression of RRGs among the gene clusters; (**F**) immunological behavior in the two gene clusters; (**G**) the results of CIBERSORT algorithm in the two gene clusters; and (**H**) comparison of TME score of three gene clusters. * *p* < 0.05; ** *p* < 0.01; *** *p* < 0.001; ns, not significant.

A total of 70 DEGs related to RRG clusters were determined for subsequent analysis (Figure S6A). Enrichment analysis disclosed immune-related pathways including T cell activation, neutrophil activation involved in immune response, natural killer cell-mediated cytotoxicity and others (Figure 4A,B). Subsequently, we performed univariate Cox analysis and consistent clustering algorithm to classify EC samples. When k = 2, the clustering performance is the best. Thus, all the EC cohorts were assigned into two gene clusters: genecluster A and genecluster B (Figure S6B–E). Survival curves illustrated that the survival probability of cases in genecluster A was higher (Figure 4C). These two gene clusters also had different clinical characteristics. The two gene clusters showed significant differences in RRG expression (Figure 4D).

Subsequently, we further investigated immunological behavior in the two gene clusters. Results of ssGSEA showed that the infiltration level of immune cells such as activated B cells, activated CD4 T cells, activated CD8 T cells and natural killer cells was higher in group A (Figure 4E,F). The results of the CIBERSORT algorithm also showed that the adaptive infiltrating immune cells, such as CD8 T cells, CD4 T cells, M0 macrophages and M1 macrophages, had a higher fraction scale in genecluster A (Figure 4G). Furthermore, patients of genecluster A had higher TME scores (Figure 4H). The expression of immune checkpoints was higher in genecluster A. The expression level of HLA between the two gene clusters was also higher in genecluster A. Classical biological pathways were more prominent in genecluster A. Based on these immune characteristics, the tumors of patients in genecluster A correspond to “hot” tumors, while genecluster B corresponds to “cold” tumors (Figure S7). Clinical correlation analysis showed that patients in both gene clusters had older age and higher grade. In terms of the clinical stage, patients in genecluster A presented lower stage. In addition, the histological type of EC cases in genecluster A was mainly endothelial, while genecluster B was mixed and serous (Figure S8).

### 3.5. Development and Validation of the RBS

**Figure 5:**
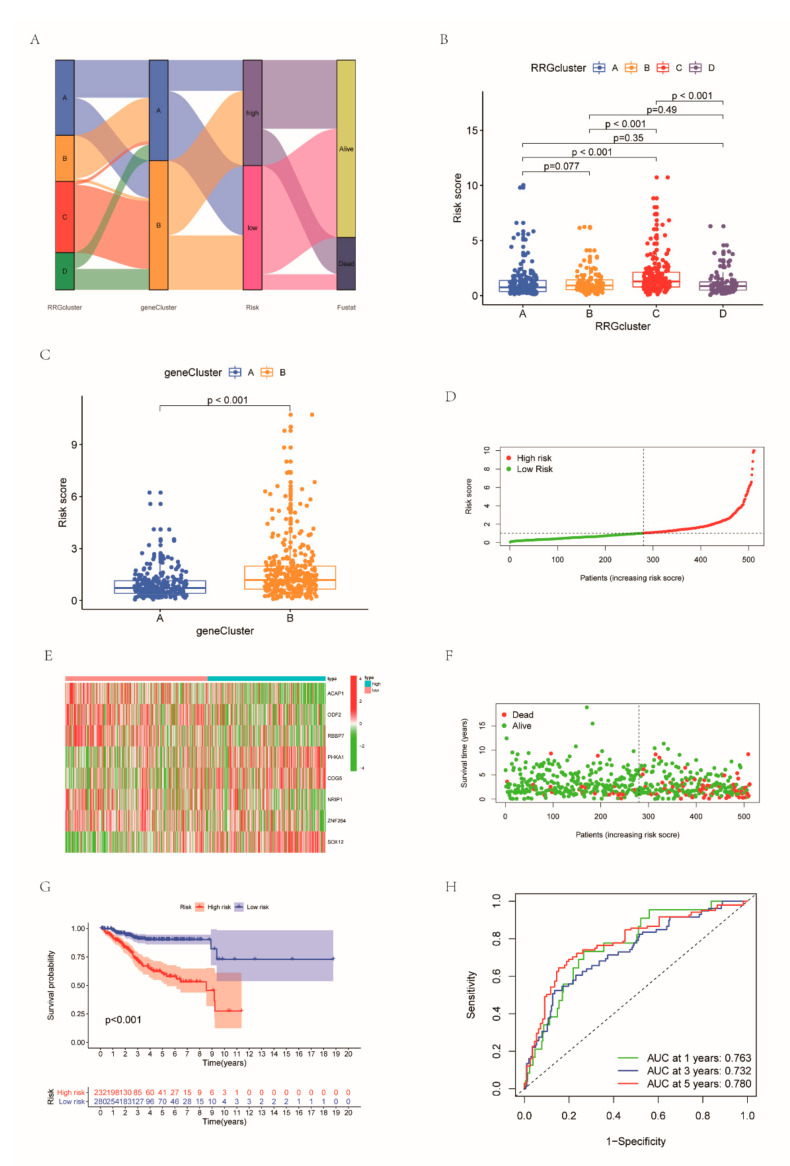
Construction of the RRG-based signature: (**A**) Sankey plot of subtype distributions in groups with different RRG scores and clinical outcomes; RRG score of the gene clusters (**B**) and RRG clusters (**C**); (**D**) distribution of risk score between two groups; (**E**) different expression of the eight RRGs between two groups; (**F**) scatter plot applied to show the fustat; (**G**) the K-M survival curve highlighting the greater survival probability in the high group; and (**H**) ROC achieved from the RRG score.

Based on these DEGs, we created an RRG scoring system to measure the prognosis of individual EC patients. Figure 5A visually shows the distribution of patients in RRG clusters, gene clusters and RRG score groups. Based on the previous screening results, LASSO regression analysis retained 12 RRGs in light of the minimum partial likelihood deviation (Figure S9A,B). Subsequently, we employed multivariate Cox analysis on these 12 genes, and finally obtained 8 genes. The risk formula was structured as follows: risk score = (−0.3374 × ACAP1) + (−0.5851 × ODF2) + (−0.3704 × RBBP7) + (0.5986 × PHKA1) + (0.668 × COG5) + (−0.2790 × NRIP1) + (−0.3796 × ZNF264) + (0.2772 × SOX12). The RRG scores of different RRG clusters and gene clusters were significantly different (Figure 5B,C). The risk scores of RRGcluster C and genecluster B were highest. We grouped patients into two risk score groups (Figure 5D). The expression patterns of eight genes in the two subgroups are shown in Figure 5E. A scatter plot shows the fustat of patients (Figure 5F). Survival curves highlight the greater survival probability in low-risk patients (Figure 5G). The AUC values of ROC curves for 1-year, 3-year and 5-year survival rates were 0.743, 0.743 and 0.773, respectively (Figure 5H). Then, we used the GEO dataset and all the cohorts were used as a testing set to verify the above results (Figure S10).

### 3.6. Comparison of the Risk Score of Different Clinical Characteristics and Stratified Analysis 

As shown in Figure S11, the risk score corresponding to older age, worse survival status, more advanced stage and more serious historical type was higher. Cox regression of RRG score and some clinical characteristics (age, histological type, grade, stage) found that our proposed signature was an independent prognostic parameter (Table S1). Furthermore, regardless of grade, stage and historical type, the outcome of the high-risk group was significantly more dismal than that of the low-risk group. 

### 3.7. Estimation of TME on the Basis of the RRG Score

**Figure 6:**
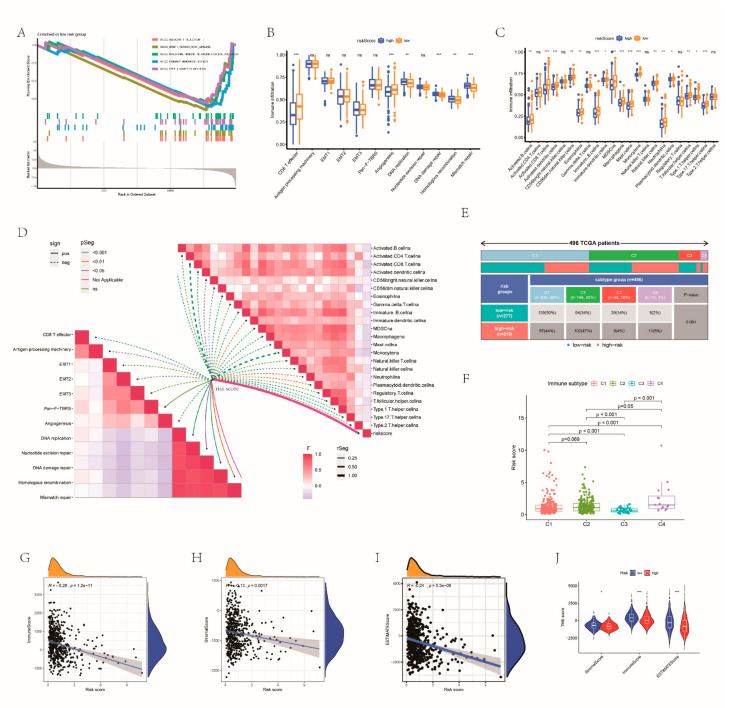
Estimation of TME on the base of the RRGs GSEA of low RRG score group (**A**). The ssGSEA score and immune infiltration score of the two groups (**B**,**C**). (**D**) The association between different immune cells and risk score and the association between risk score as well as classical biological pathway score. (**E**) Immune subtype classification of each risk group in 496 TCGA patients. (**F**) The risk scores of 4 immune subtypes. The relationships between RRG score and stromal cells (**G**), immune cells (**H**) and estimated score (**I**). (**J**) Comparison of the TME score between the RRG clusters. * *p* < 0.05; ** *p* < 0.01; *** *p* < 0.001; ns, not significant.

**Figure 7:**
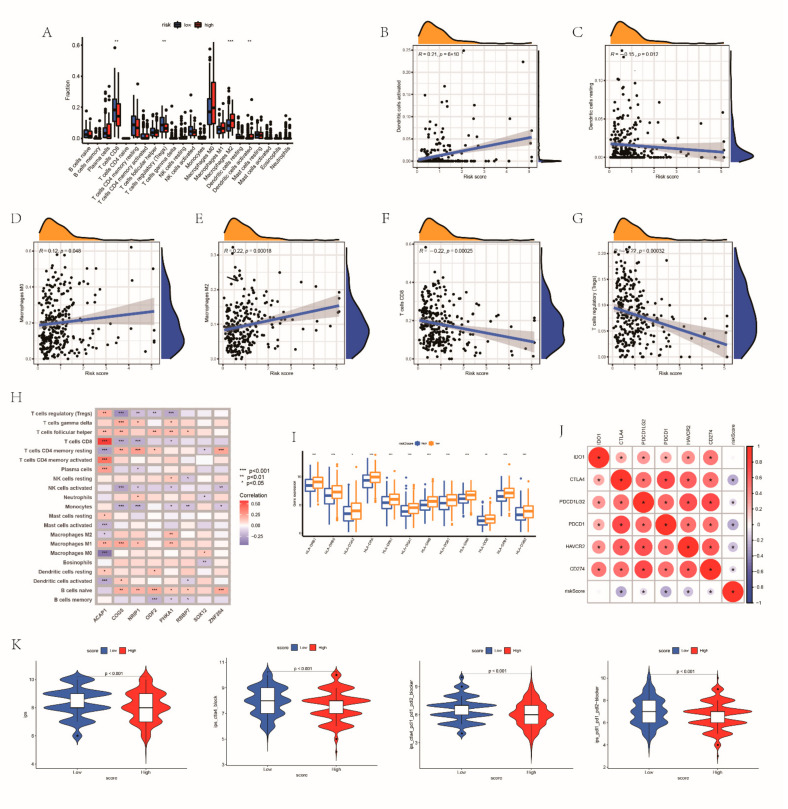
Immune infiltration characteristics between the two subgroups: (**A**) the immune cell abundance of the groups; (**B**–**G**) the relationship between 8 genes and immune cell abundance; (**H**) the correlations between RRGs and immune cell abundance; (**I**) the expression level of HLA between the groups; (**J**) the associations between immune checkpoints and risk score; and (**K**) the differences of IPS score in patients with different risk. * *p* < 0.05; ** *p* < 0.01; *** *p* < 0.001; ns, not significant.

To further analyze the TME of the different groups, the GSEA method was conducted. We noticed that the low-risk group was concentrated in immune-related processes (Figure 6A). As expected, significantly abundant immune pathways, such as CD8 T effector, were detected in patients with a low risk score (Figure 6B). Figure 6C illustrated the immune cell landscape of both groups. The low-risk group had higher infiltration levels of activated CD8 T cells, macrophages, monocytes and type 17 T helper cells. There were negative correlations between RRG score and almost all immune cells. The relevance between risk score and immune-related biological pathways is also shown in Figure 6D. In order to further analyze the characteristics of the TME of the two groups, 496 TCGA patients were assigned into different immune subtypes. The most common subtypes in both groups were cluster 1 and cluster 2. The risk scores of the cluster 1 and cluster 2 immune subtypes had no obvious difference, but they were significantly higher than the risk scores of the cluster 3 and cluster 4 subtypes (Figure 6E,F). The risk score had a negative correlation with TME cells (Figure 6G–I). The TME scores of the groups were also compared (Figure 6J). Similarly, the stromal cells and estimated scores between the two groups are different. Great differences existed in the expression levels of CD8 T cells, Tregs, M2 macrophages and dendritic cells between the two risk groups (Figure 7A). The correlation between RRG score and immune cell abundance is shown in Figure 7B. RR score had a positive correlation with activated dendritic cells activated, M0 macrophages and M2 macrophages, and had a negative correlation with resting dendritic cells, CD8 T cells and Tregs (Figure 7C–G). The associations between immune cells and the expression levels of eight RRGs are shown in Figure 7H. HLA expression was also higher in patients with lower risk scores (Figure 7I). Also, we calculated the association between immune checkpoints and RBS (Figure 7J). The IPS score of patients with a low risk score was higher (*p* < 0.05) (Figure 7K). Thus, we inferred that high-risk EC belongs to cold tumors and may receive less benefit from immunotherapy.

### 3.8. Relationships between RRG Score and Tumor Stem Cells as well as TMB

**Figure 8:**
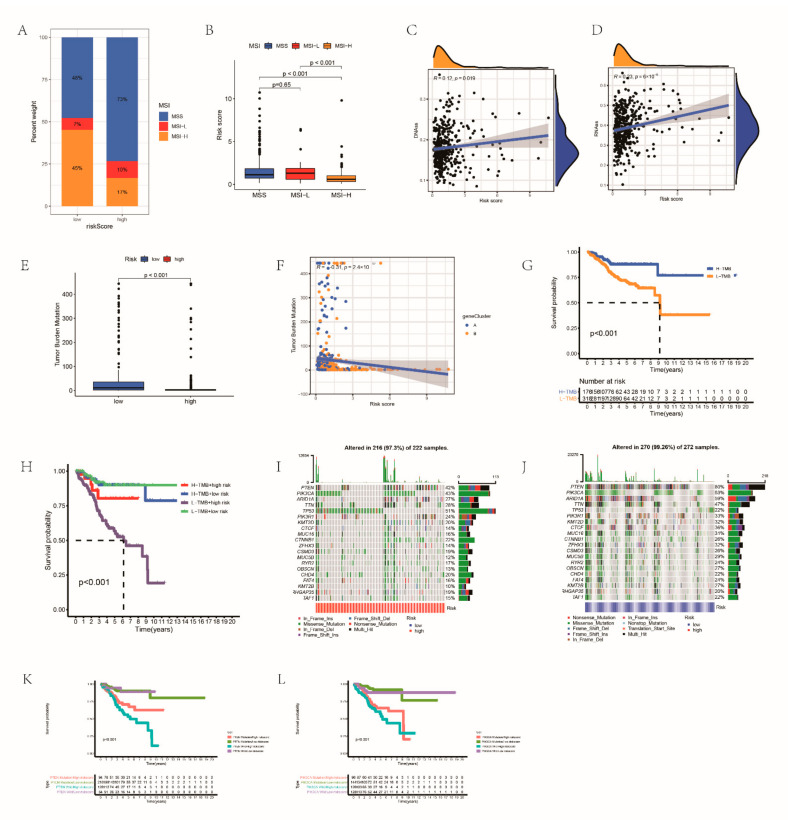
Calculating the effect of immunotherapy: (**A**,**B**) correlations between RRG score and MSI; (**C**,**D**) the result of linear relationship between RRG score and CSC index; (**E**) comparison of the TMB between the groups; (**F**) the associations between TMB and RRG score; (**G**) the OS of different TMB patients; (**H**) the survival probability of patients with different TMB and risk; the mutation information of genes with high mutation frequency in the high- (**I**) and low-risk groups (**J**); (**K**) the survival probability of patients with different PTEN mutation and risk score; and (**L**) the survival probability of patients with different PIK3CA mutation and risk score.

6×10^−4^ In the high RRG score group, the microsatellite stability status had a high percent weight, while the RRG score of high MSI was lower (Figure 8A,B). The interaction between tumor stem cells and immune cells can promote the progression of various cancers. We analyzed the regulatory role of RRG score in EC stem cells by analyzing RNAss and DNAss. RRG score was significantly positively correlated with two indicators, indicating that RRG cells with higher scores had more obvious stem cell characteristics and lower cell differentiation (Figure 8C,D).

Increasing evidence showed that higher TMB was related to more neoantigens in the tumor, and increased the susceptibility of patients to immunotherapy [38]. Therefore, we next comprehensively evaluated the distribution of TMB in the two groups. There was a significant negative correlation between risk score and TMB (Figure 8E,F). The outcome of the low TMB group was dismal (Figure 8G). At the same time, low TMB together with high risk means a low survival rate (Figure 8H). Next, we calculated the distribution of somatic mutations in risk groups (Figure 8I,J). The mutation frequency of both groups was very high (low-risk group: 99.26%, high-risk group: 97.3%), and the mutation frequency of PTEN and PIK3CA was the highest. Further evaluating the OS in the case of PTEN and PIK3CA mutations, we found that the OS was significantly lower when PTEN and PIK3CA mutated and lower combined with high-risk (Figure 8K,L). Figure S12A indicated the distribution of GISTIC scores calculated in the light of the frequency and amplitude of gain and loss on all chromosomes in the two groups. Focal amplification and deletion of different chromosome regions were detected in both groups (Figure S12B,C).

### 3.9. Analysis of Drug Sensitivity

We selected chemotherapeutic drugs commonly used in EC treatment to calculate the sensitivity of patients to these drugs. The IC50 of cisplatin and doxorubicin was lower in high-risk patients, while the IC50 of methotrexate was lower in the low-risk subgroup (Figure S13A). The correlation between eight genes and different drugs is shown in Figure S13B. These results suggest that the eight genes are associated with drug sensitivity.

### 3.10. Development of Nomograms for Survival Prediction 

According to Table S1, risk score, historical type and stage are independent prognostic factors, so we incorporated risk score, histological type and stage to establish a nomogram (Figure S14A). The C index of RBS was higher than other clinical features, and the C index was highest when considered together with other clinical factors (Figure S14B). The AUC of the RBS was generally higher than that of historical type and stage, and the prediction effect was better. The nomogram that combined RRG risk, historical type and stage had a better prediction effect (Figure S14C–E). DCA showed that the combination of RBS and clinical characteristics displayed a higher benefit in predicting the prognosis of patients with EC patients (Figure S14F–H). The subsequent calibration diagram showed that the nomogram has great performance compared with the actual situation (Figure S14I).

### 3.11. RRG Score Is a Novel Predictor for EC Patients

To better demonstrate the predictive ability of our model, we screened four EC prognostic models from the published literature and compared them with our model. In order to make them comparable, according to the four previously established models, we also applied multivariate analysis to calculate the risk value and prognosis evaluation of each data set. The survival analysis indicates that the prognosis of people with a high RRG score was much worse in all four models (Figure S15A). The AUC values of the models were lower than our proposed RBS (Figure S15B). Therefore, we believe that they are inferior to our model in predicting prognosis. As shown in Figure S15C, obviously, the C index of all prognostic features of our model was the highest, which was 0.678 (Figure S15C). Our genetic characteristics perform best around the 11th year. This suggests that our model is the best predictor of 11-year survival compared to other models (Figure S15D).

## 4. Discussion

TME-related redox can regulate redox balance by forming an antioxidant defense system [39], which play an anti-tumor role to a certain degree [40]. In recent years, many researchers have devoted themselves to analyzing tumor treatment strategies based on redox. However, the regulatory mechanism of redox in tumors needs to be further analyzed.

In this academic research, we unearthed the genetic characteristics and clinical potency of RRGs in EC. RRGcluster B had the most abundant immune infiltration, significant immune activation characteristics and the highest TME score and expression level of immune checkpoints. This indicates that RRGcluster B is roughly equivalent to “hot” tumors, which will have a higher response to immunotherapy. Patients belonging to RRGcluster C had the shortest OS. At the same time, RRGcluster C had the lowest TME score and the highest tumor purity. The enrichment degree of RRGcluster D is significantly different from that of the other three RRG clusters. The enrichment degree is low in some processes related to immune activation. Based on the above analyses, we consider RRGcluster C and RRGcluster D as “cold” tumors, which are less likely to be benefit from immunotherapy. Furthermore, patients in genecluster A have milder clinicopathological features, higher levels of immune cell infiltration, a higher expression of immune checkpoints and, not surprisingly, a higher survival probability. We believe that tumors of patients in genecluster A correspond to “hot” tumors. We further determined two gene clusters based on 70 DEGs related to RRG cluster. Survival analysis uncovered that there was a significant difference in OS among the two gene clusters, indicating that the gene-related subtype derived from RRG cluster is also effective in predicting patient prognosis.

The RBS consists of ACAP1, ODF2, RBBP7, PHKA1, COG5, NRIP1, ZNF264 and SOX12. ACAP1 is closely bound up with the level of immunocyte infiltration, immune regulators and chemokines, and has been used in the prediction of clinical outcomes with several tumors, including gynecologic tumors [41,42,43]. As for ODF2, we have also explored its role in EC [44]. RBBP7 functions as a subunit of a variety of chromatin-related complexes in epigenetic regulation and is associated with many cancers [45]. PHKA1 is a regulator of glycogen metabolism [46], but its role in cancer has been less studied. COG5 is involved in metastatic inflammation and cartilage formation and can represent a prospective treatment target for chondrosarcoma [47]. NRIP1 exerts a carcinogenic effect in various solid tumors [48,49,50]. Furthermore, NRIP1 is a classical mutated gene in EC cell lines [51], and its overexpression is a common cause driving EC and advanced myometrial invasion [52,53]. RNA editing levels of ZNF264 are greatly correlated with the clinical outcome of EC [54]. Overexpression of SOX12 was associated with a loss of tumor capsule [55,56]. Therefore, the eight genes are potential signature to assess the prognosis of EC. Additionally, survival analysis indicated that there were 34 RRGs that had notable prognosis values in EC. Of these genes, 17 are potential risky genes since their high expressions are associated with a favorable prognosis of EC cases. Meanwhile, the other 17 genes are potential protective genes given that their high expressions are associated with a poor prognosis of EC patients.

Stratified survival analysis under different pathological characteristics disclosed that patients with a high RRG score had a dismal survival outcome. The insignificant difference in the testing set may be owing to the small sample size of this subgroup.

In recent years, the TME has been recognized as an overwhelming contributor to tumor progression [5]. Previous reports have focused on TME reactive therapy to achieve an accurate targeted treatment of cancer [57,58]. Immune and stromal scores have been found to be positively correlated with the clinical features and fustat of EC [59]. Compared with the high-risk group, low risk patients had a higher level of immune cell infiltration, such as activated CD8 T cells, immune B cells, macrophages, monocytes and type 17 T helper cells, which means strong antitumor immunity. CD8 + T cells are an inhibitor of tumors [60,61] and are the strongest predictor of EC recurrence [62,63,64]. B cells also have a vital effect on anti-tumor response. Their main function is to identify specific antigens with cell surface immunoglobulin or B cell receptors [65,66]. As one of the most important components in the TME [67], macrophages can support tumor viability and survival by secreting immunosuppressive factors, cytokines and growth factors [68]. It has been demonstrated that increased macrophage infiltration is associated with an adverse prognosis in EC [69]. The role of helper T cells in EC has not been confirmed. In addition, regulatory T cells, which inhibit immune activity and antitumor immune response, were enriched in cohorts with low RRG scores [70,71]. This is consistent with our findings that patients in RRGcluster B, genecluster A and the low RRG score group have higher immunocyte infiltration and a better prognosis. Moreover, stromal and estimate scores were higher in the low RRG score group, suggesting they belong to hot tumors and have a poor response to immunotherapy. Cold tumors imply a large percentage of immunosuppressive cells within the tumor [72]. Our results showed that the high-risk group, RRGcluster C, RRGcluster D and genecluster B belong to cold tumors. This indicates that the high RRG score group is prone to a lack of immune surveillance function, which facilitates tumor immune escape [73]. 

At present, the options of EC treatment are limited. Immunotherapy, especially checkpoint inhibitors, has made exciting progression in gynecological malignancies [74]. Consistent with previous conclusions, there is a negative correlation between RRG score and the expression level of common immune checkpoints. IPS score has been reported as being able to reflect the response to immunotherapy [75]. IPS score in the low-risk population was higher, suggesting this subgroup had higher immunogenicity. According to the above results, EC patients with low RRG scores may have a better response to immune checkpoint therapy. The PD-1 inhibitor pembrolizumab has been identified as an effective treatment for EC patients [76]. TMB is considered to be another essential predictor of immunotherapy. Various tumor cases indicated that a higher TMB score means a favorable outcome after immunotherapy [73], which is consistent with the above results. Furthermore, there is evidence that high MSI patients are more likely to benefit from immunotherapy [77]. This further confirmed that the lower RRG score group may benefit from immunotherapy.

RRG score has a positive correlation with RNAss and DNAss, which means that high-risk people are more prone to EC growth [78]. Consistent with previous studies, PTEN and PIK3CA are common genetic aberrations in EC [79], and play a central part in EC development [80,81]. The CNV frequency was higher in patients with a high risk score and the subgroup with a high copy number had the worst prognosis [82].

Cisplatin and doxorubicin have been identified as effective drugs for EC [83]. Methotrexate is also effective in EC patients [84]. Here, we found that the IC50 of cisplatin and doxorubicin was lower in high-risk patients while IC50 of methotrexate was lower in low-risk patients. The results showed that high-risk cases are more sensitive to cisplatin and doxorubicin and low-risk patients are more sensitive to methotrexate, indicating that our proposed signature could serve as a potential predictor for chemosensitivity and offer a valuable reference for individualized treatment. 

Finally, by integrating stage, historical type and RRG score, we constructed a nomogram to more intuitively show the role of these factors in predicting EC and to improve the clinical application of RRG score. Four EC prognostic risk models were selected from published articles and compared [85,86,87,88]. 

The current research does have some limitations. Firstly, all conclusions come from the processing and analysis of public database data, and there is a lack of clinical data and experimental research to verify the results. In the future, we need to further collect enough EC cases and conduct a large number of prospective clinical analyses to ensure the effectiveness of the RBS in clinical application. The mechanism of some of these genes in EC has not been reported and needs to be further explored.

## 5. Conclusions

We built a prognostic model of EC on the basis of eight RRGs and verified its good prediction performance. This paper comprehensively analyzed their effects on the TME, clinical characteristics and prognosis, and determined their therapeutic effects in targeted therapy and immunotherapy, which can help determine the prognosis and offer new therapeutic targets for patients. It offers a new direction for guiding the personalized treatment strategy of EC patients.

## Data Availability

The datasets supporting the conclusions of this article are included within the article.

## Supplementary Materials

The following supporting information can be downloaded at: https://www.mdpi.com/article/10.3390/cancers14143383/s1, Figure S1: The analysis process of this study. Figure S2: OS differences corresponding to different gene expression level. Figure S3: Consensus clustering analysis. Figure S4: GSVA of biological pathway between four different groups. Figure S5: GSVA of biological pathway between four different clusters. Figure S6: Consensus clustering analysis. Figure S7: Gene subtype identification based on DEGs. Figure S8: Clinical correlation analysis of two geneclusters. Figure S9: Identifying representative candidate prognostic genes. Figure S10: Validation of the RBS. Figure S11: Clinical relation analysis and stratified analysis of the model. Figure S12: Gene mutation analysis of the model. Figure S13: Analysis of drug sensitivity. Figure S14: Establishment of a nomogram. Figure S15: Comparison of RBS with other established models. Table S1: Univariate and multivariate Cox regression analyses of the prognosis-related factors in all set.

## Abbreviations

AUC	area under curve
CNVs	copy number variations
DCA	decision curve analysis
DEGs	differentially expressed genes
EC	endometrial carcinoma
GEO	Gene Expression Omnibus
HLA	human leukocyte antigen
IC50	half maximum inhibitor concentration
IPS	immunophenoscore
LASSO	least absolute shrinkage and selection operator
MSI	microsatellite instability
OS	overall survival
PCA	principal component analysis
RMS	restricted mean survival
ROC	receiver operating characteristic
RRGs	redox-related genes
TCGA	The Cancer Genome Atlas
TIIC	tumor-infiltrating immune cells
TMB	tumor mutation burden
TME	tumor microenvironment
GSVA	gene set variation analysis
GSEA	gene set enrichment analysis
