# Supplementary material for: Crosstalk of Redox-Related Subtypes, Establishment of a Prognostic Model and Immune Responses in Endometrial Carcinoma
Source: Cancers (Basel). 2022 Jul 12;14(14):3383. doi: 10.3390/cancers14143383 (PMC9319597; doi:10.3390/cancers14143383)
Supplement: Supplementary file 1 [file cancers-14-03383-s001.zip › cancers-1770889-supplementary.pdf]

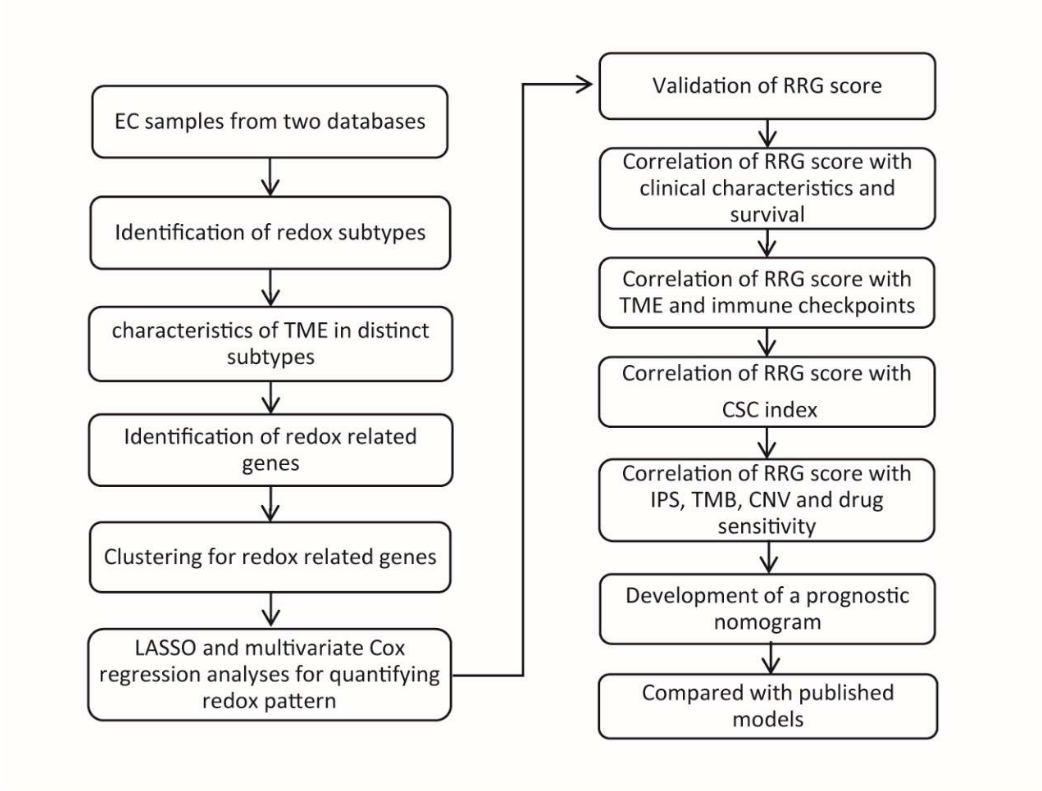

Figure S1 The analysis process of this study.

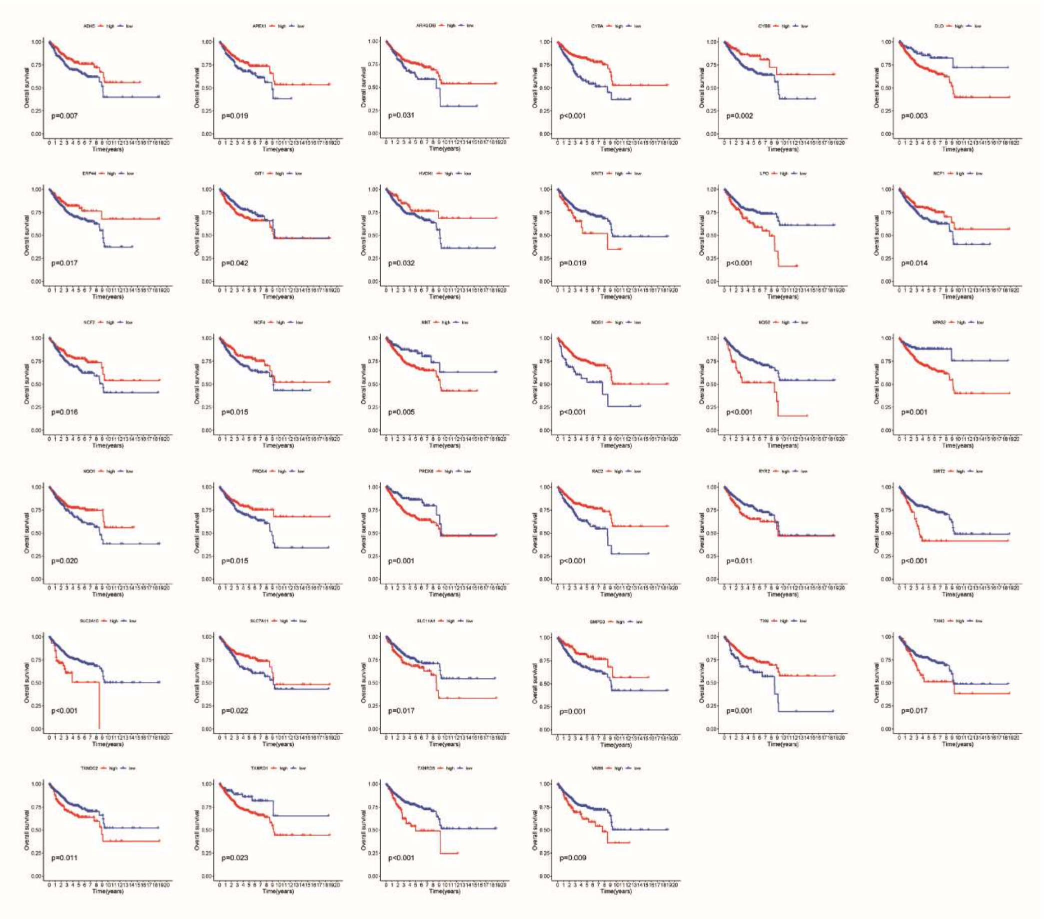

Figure S2 OS differences corresponding to different gene expression level.

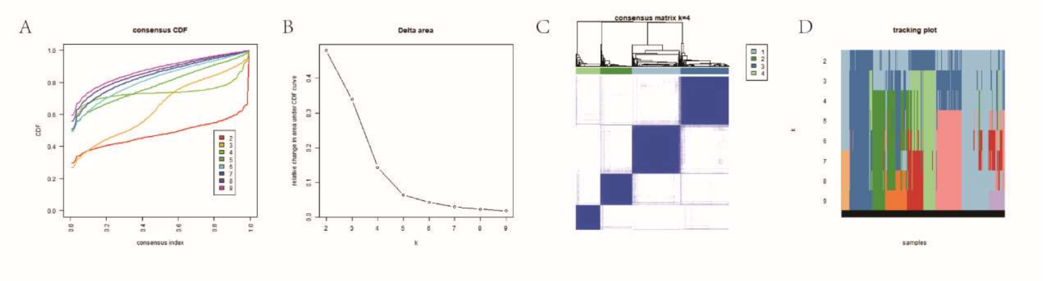

Figure S3 Consensus clustering analysis. (A) Uniform clustering cumulative distribution function (CDF), k = 2-9 (k represents the number of clusters). (B) The change of area under CDF curve with k = 2-9. (C) The samples were divided into four clusters when k=4. (D) Tracking plot of the cluster when k=4.

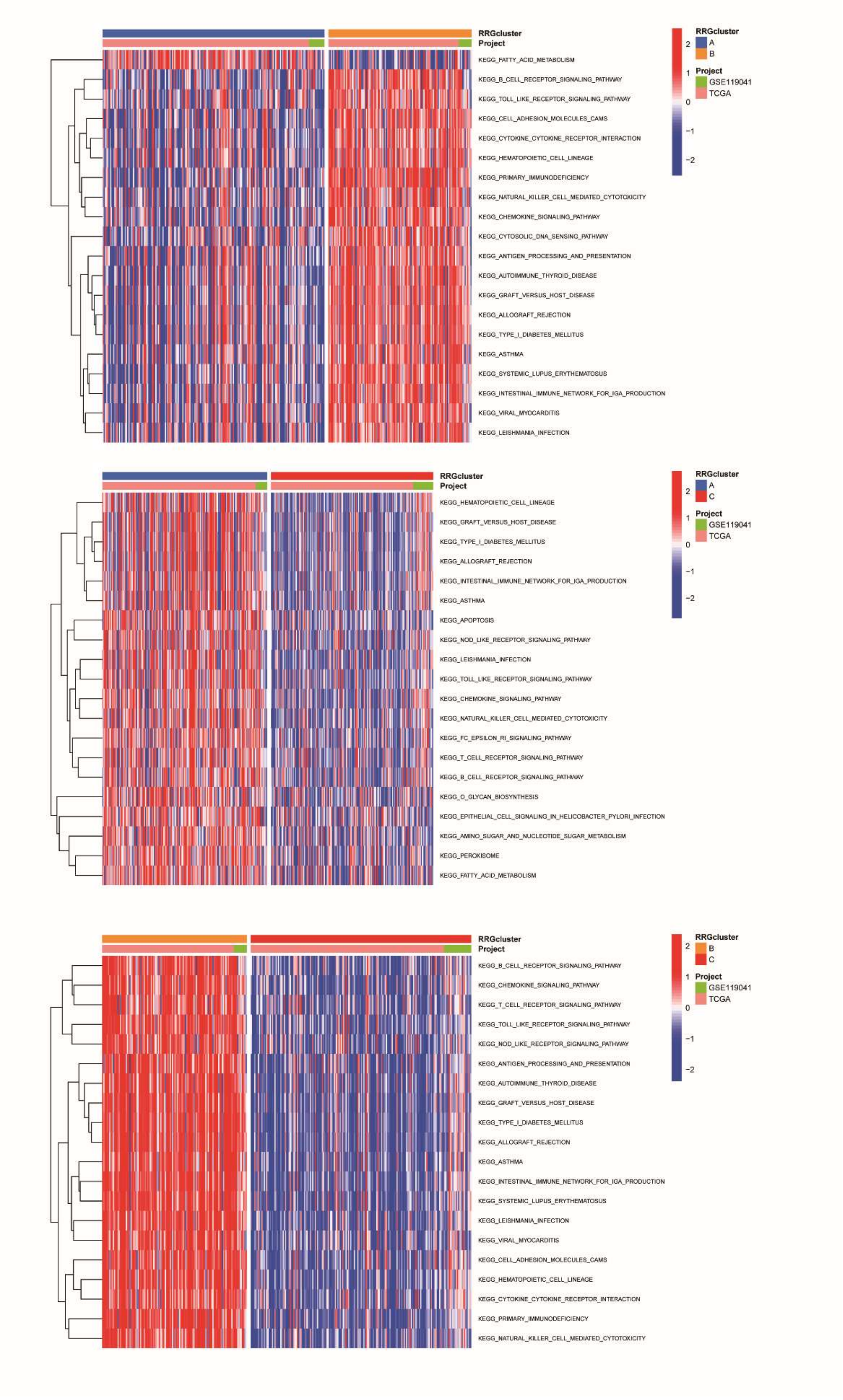

Figure S4 GSVA of biological pathway between four different groups.

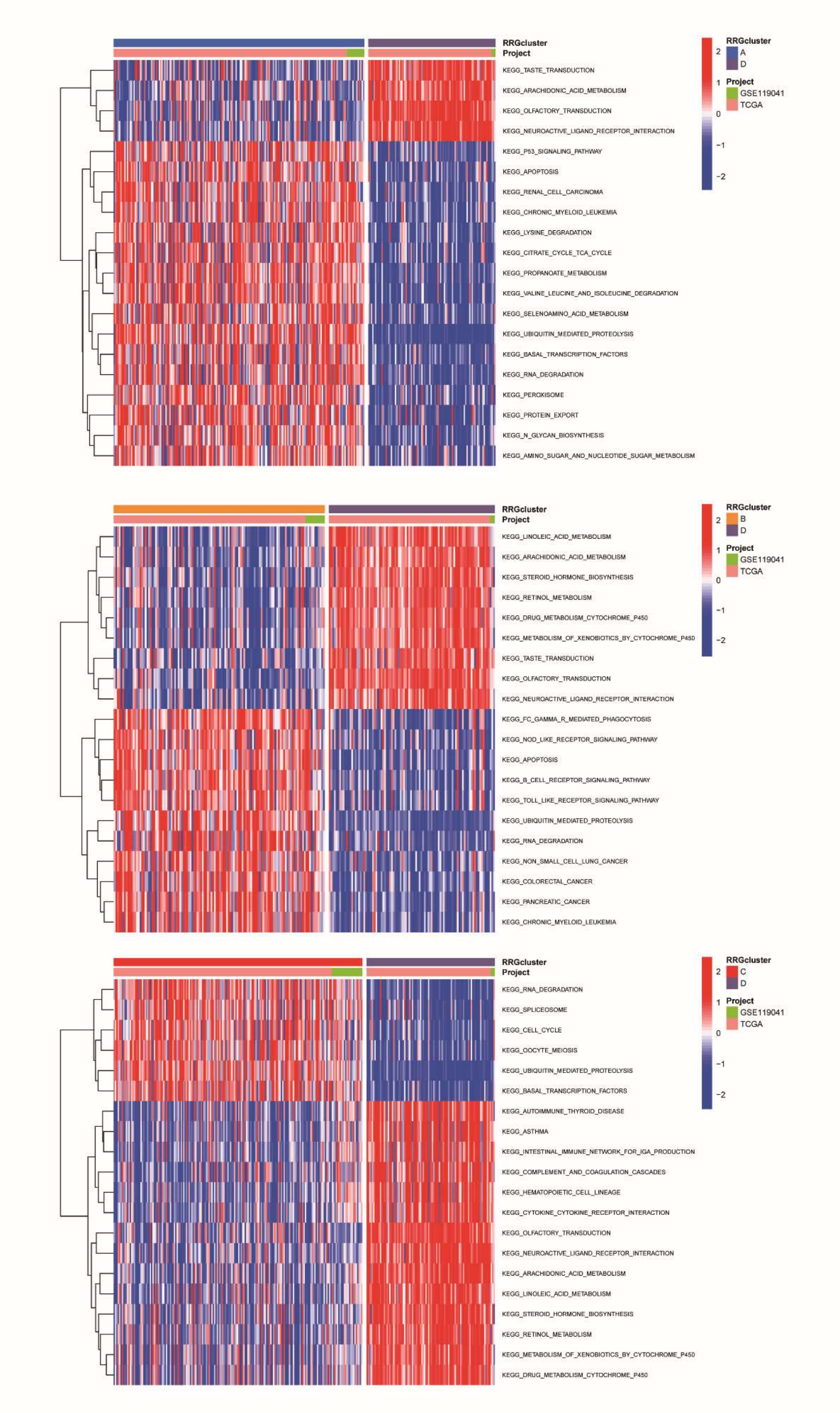

Figure S5 GSVA of biological pathway between four different clusters.

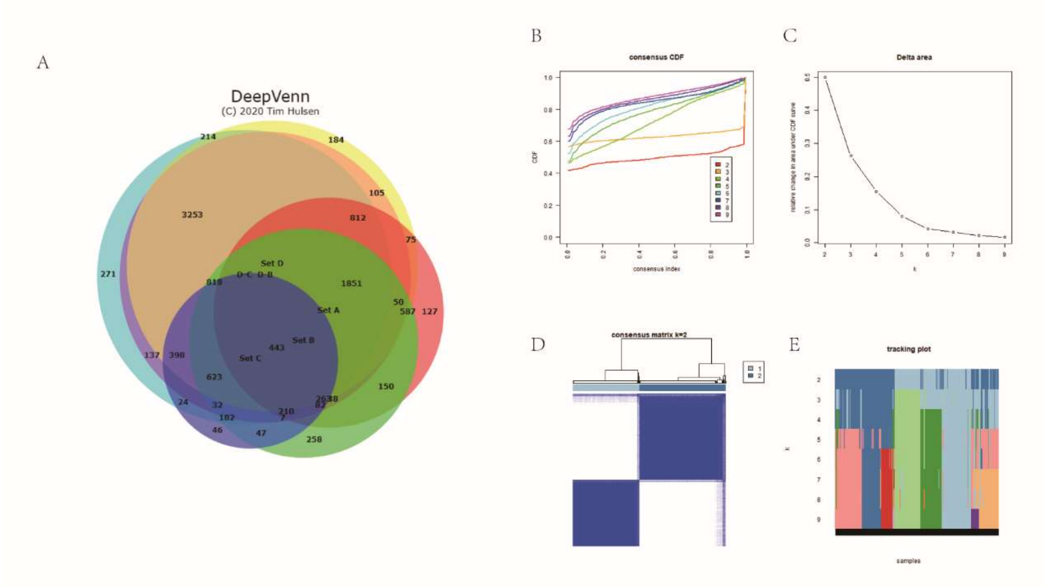

Figure S6 Consensus clustering analysis. (A)Intersection of DEGs in RRG clusters shows in venn diagram. (B) Uniform clustering CDF, k = 2-9 (k represents the number of clusters). (C) The change of area under CDF curve, k = 2-9. (D) The samples were assigned into two geneclusters when k=2. (E) Tracking plot of the cluster when k=2.

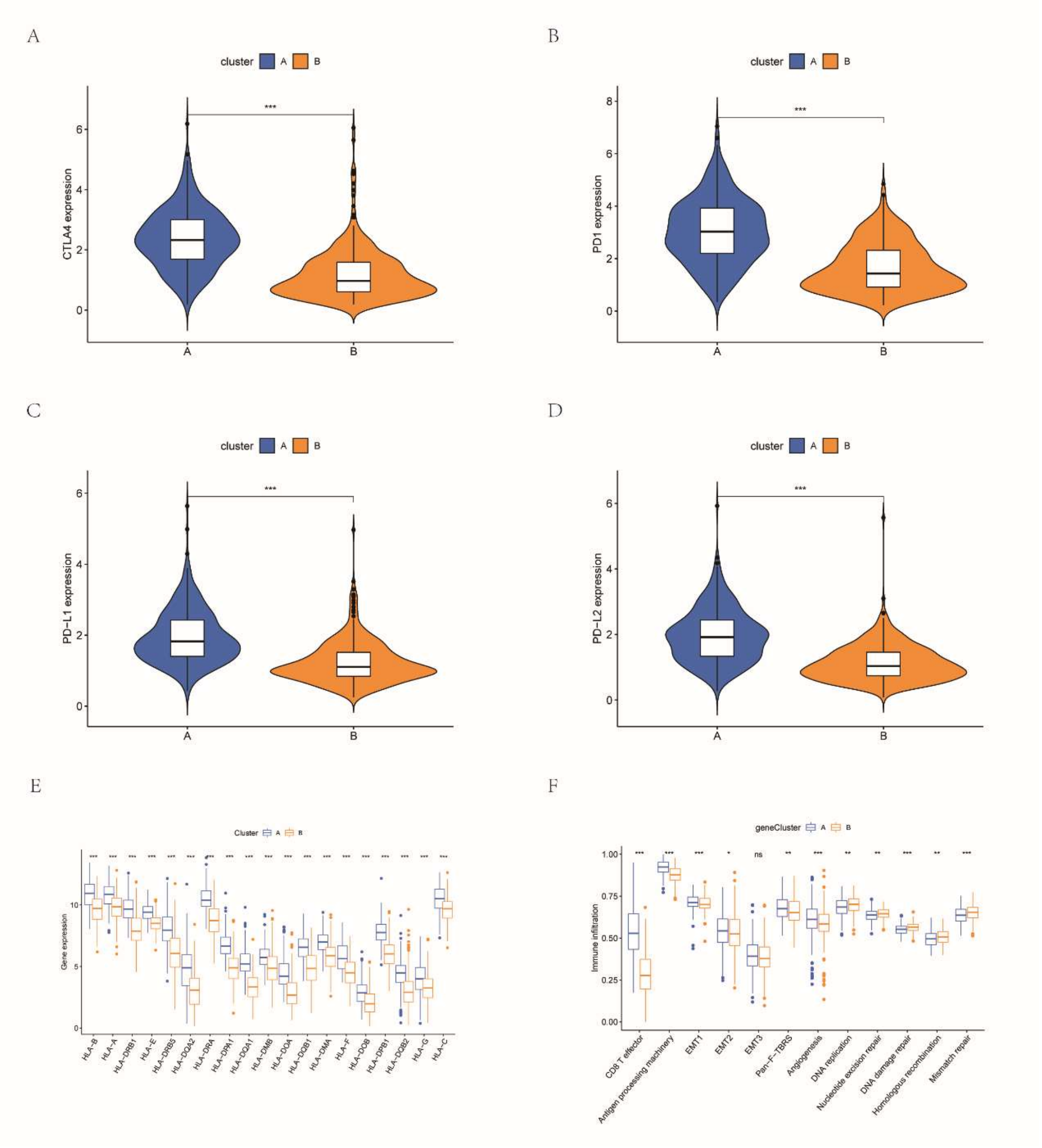

Figure S7 Gene subtype identification based on DEGs. (A-D) Expression level of immune checkpoints in two geneclusters. (E) HLA expression level of geneclusters. (F) Compare the scores of biological processes between the geneclusters.

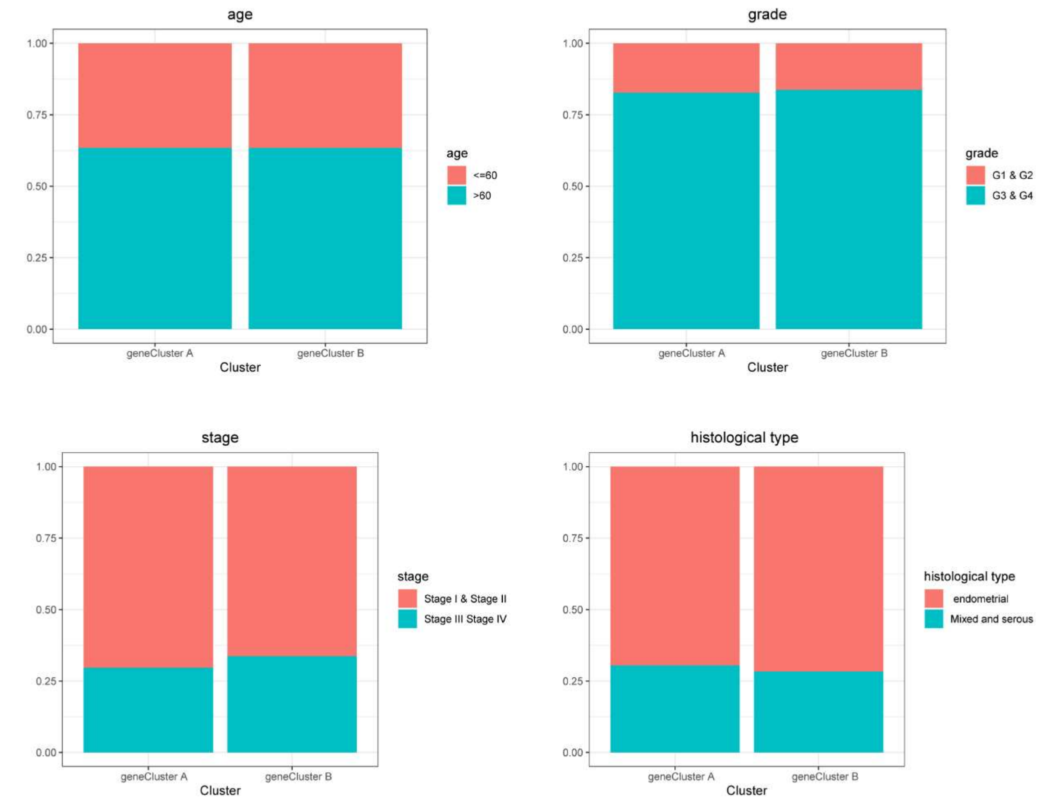

Figure S8 Clinical correlation analysis of two geneclusters.

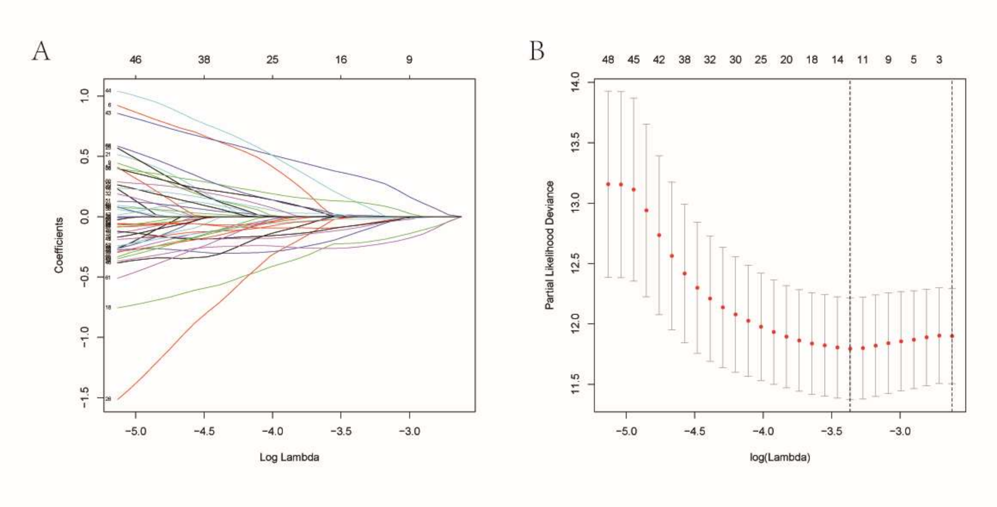

Figure S9 Identifying representative candidate prognostic genes. (A-B) LASSO regression analysis and partial likelihood deviance of candidate genes

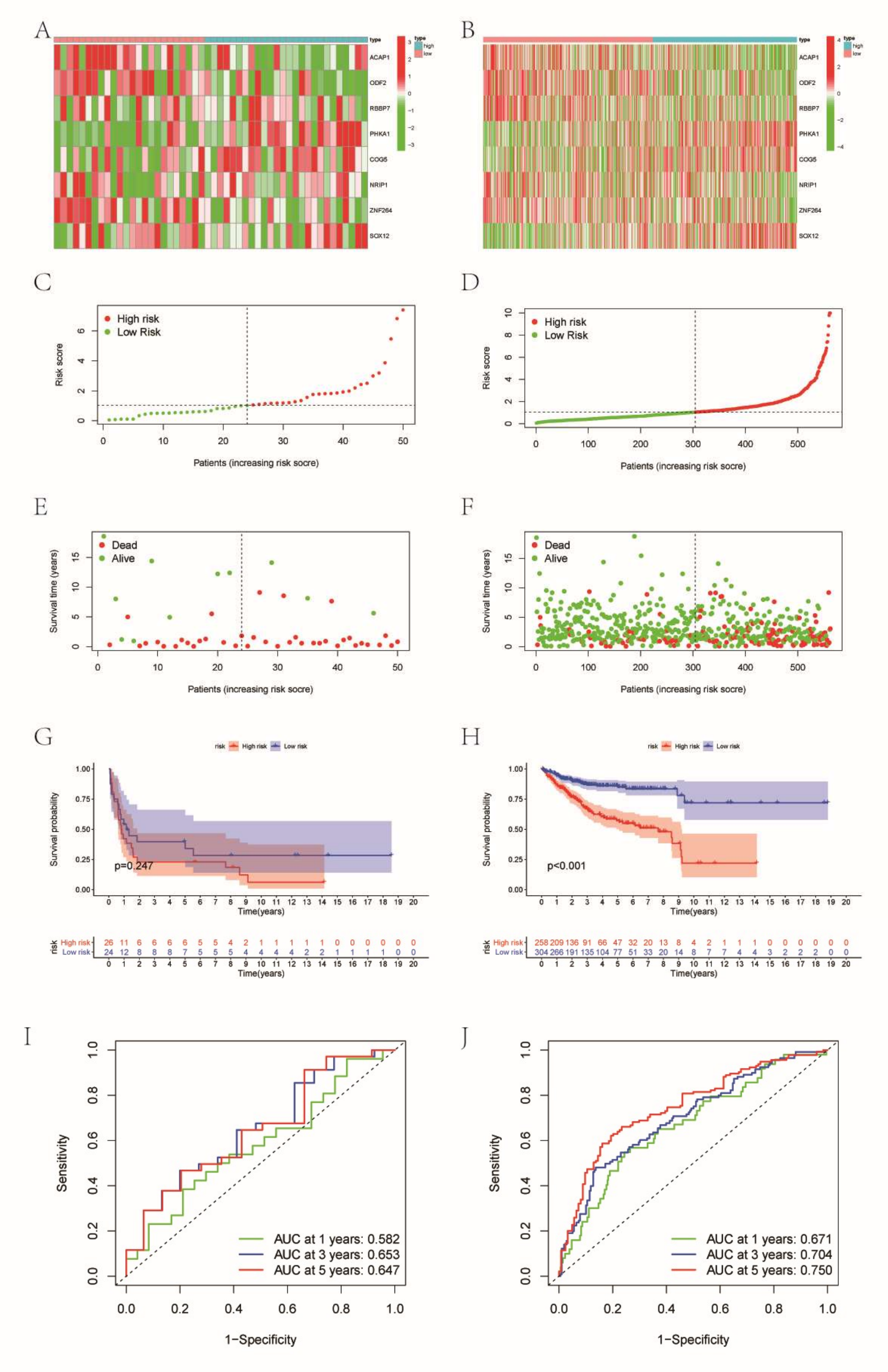

Figure S10 Validation of the RBS. The expression of the 8RRGs in GEO set (A) and all set (B). People in GEO set (C) and all set (D) were assigned

into two risk groups. Distribution of survival time in GEO set (E) and all set (F). Survival probability of the groups in GEO set(G) and all set(H). ROC curves of GEO set(I) and all set(J).

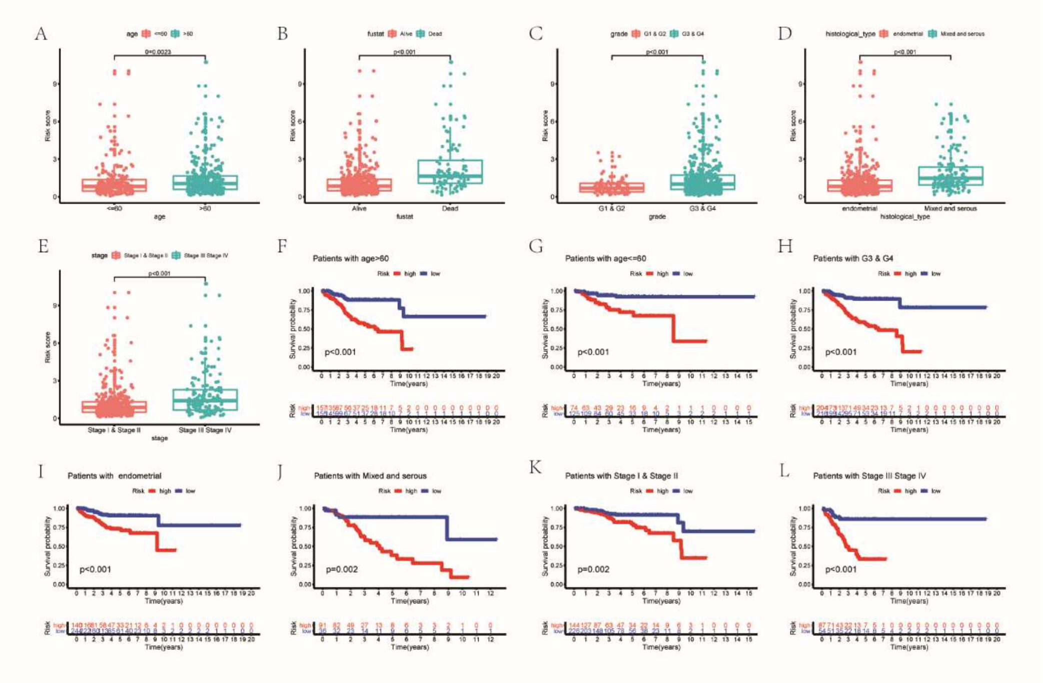

Figure S11 Clinical relation analysis and stratified analysis of the model. Comparison of risk scores of different age(A), fustat (B), grade(C), historical type(D) and stage(E). (F-L) Survival analysis of EC patients with different clinicopathology features.

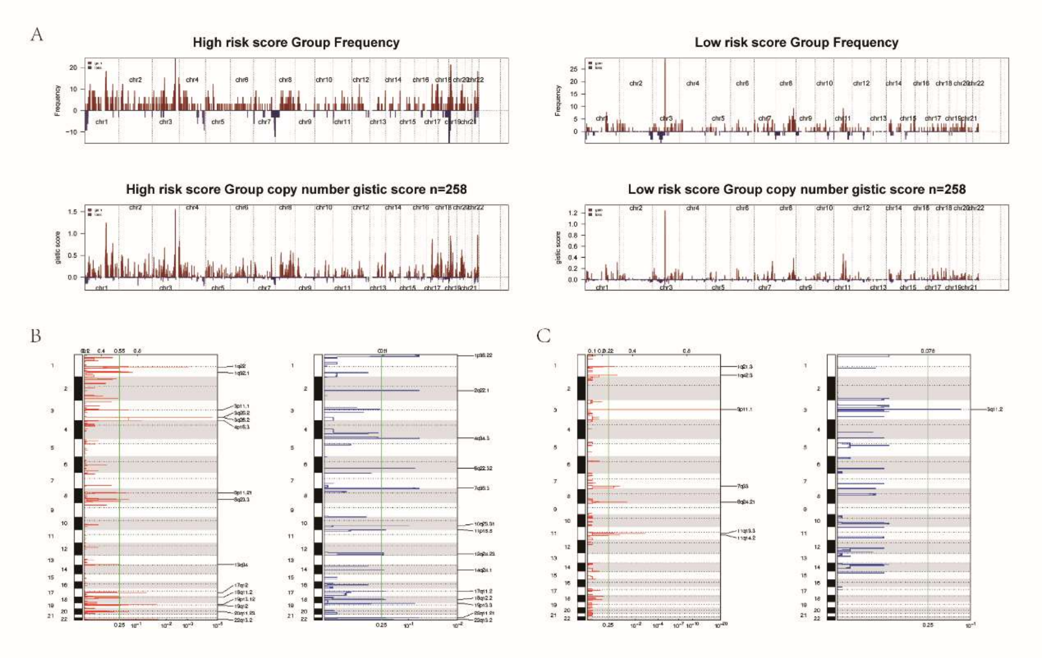

Figure S12 Gene mutation analysis of the model.

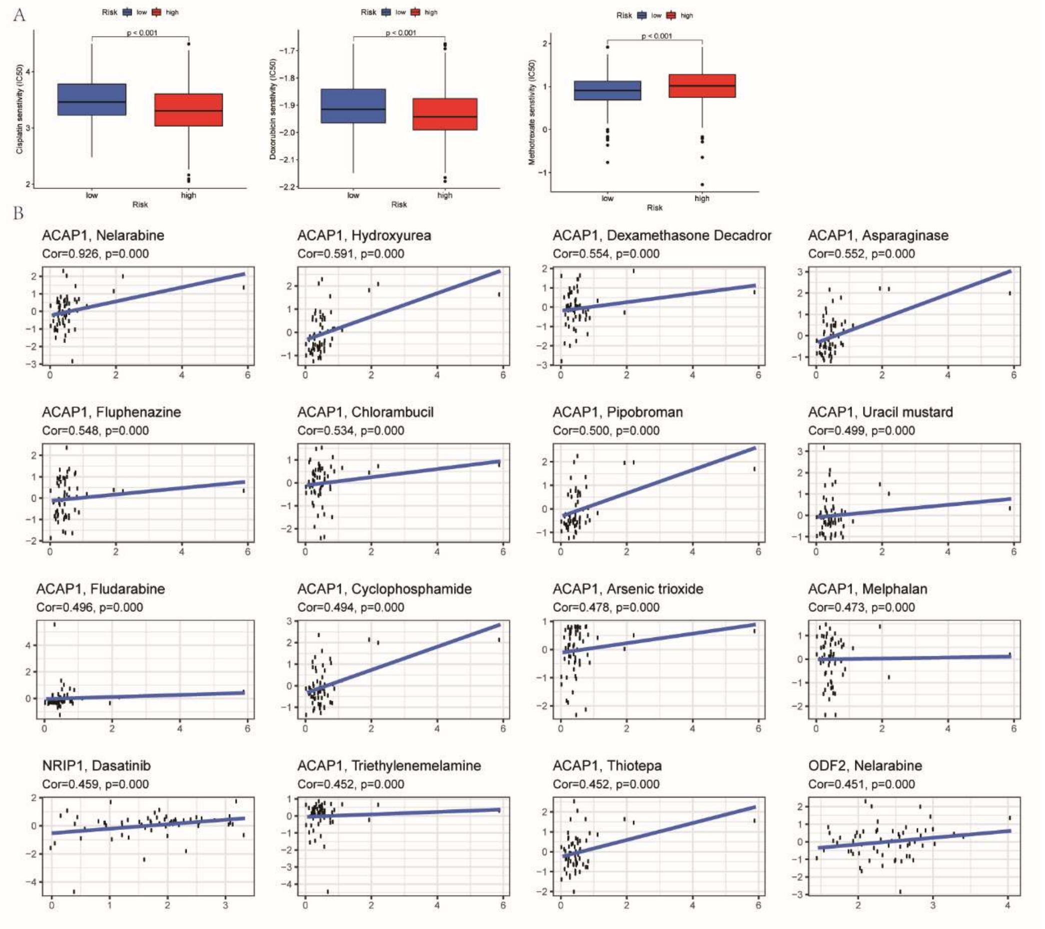

Figure S13 Analysis of drug sensitivity. (A)The difference of IC50 of 3 chemical drugs between the groups. (B) Associations between common drugs and 8 genes.

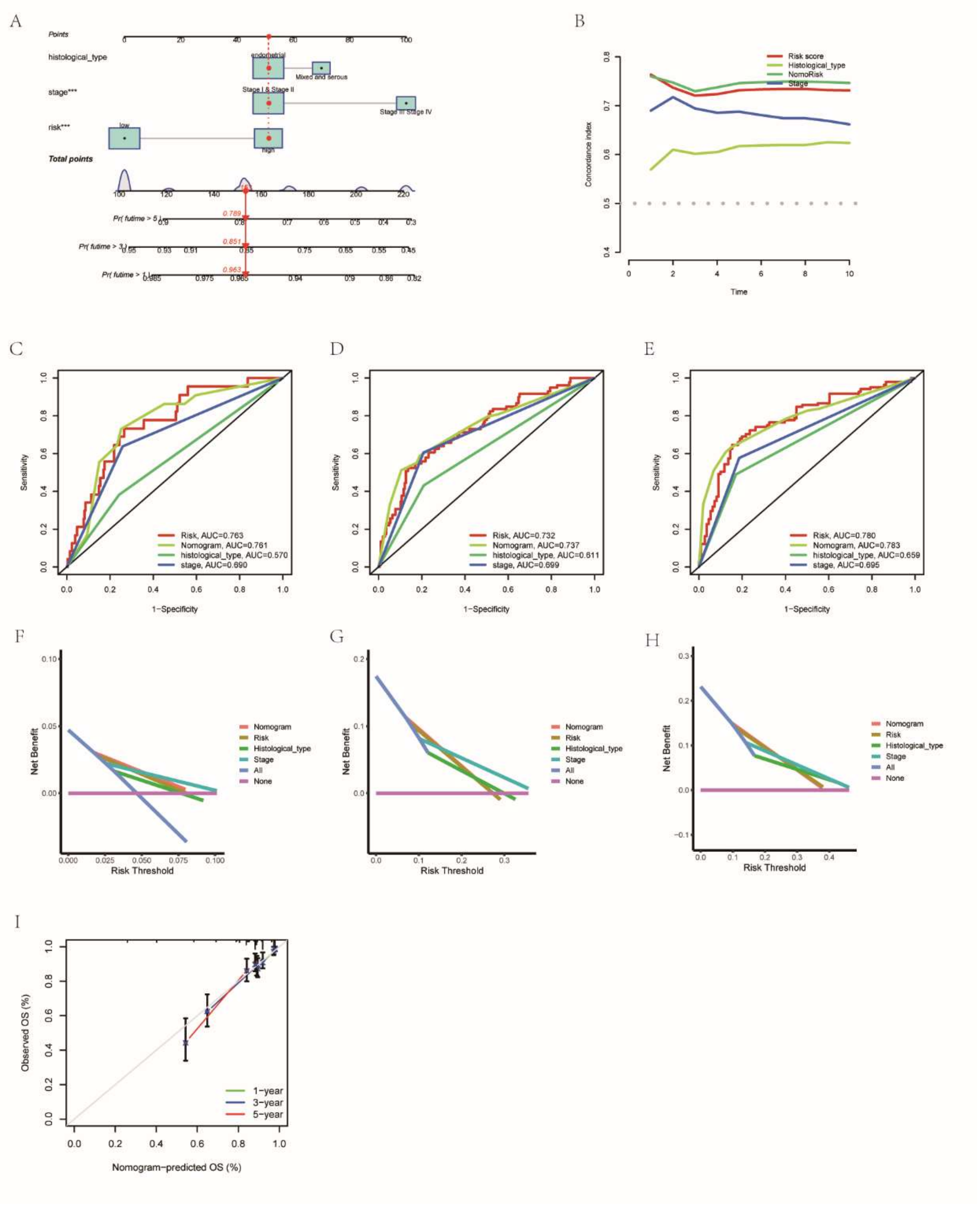

Figure S14 Establishment of a nomogram. (A) Nomograms applied to predict OS of EC patients. (B) The consistency index of prognostic factors including risk score. (C-E）ROC curves of different years.(F-H) DCA

analysisused to calculate net benefit of 1 -, 3 -, and 5-years (I) Nomogram cali-bration curve of 1 -, 3 -, and 5-years.

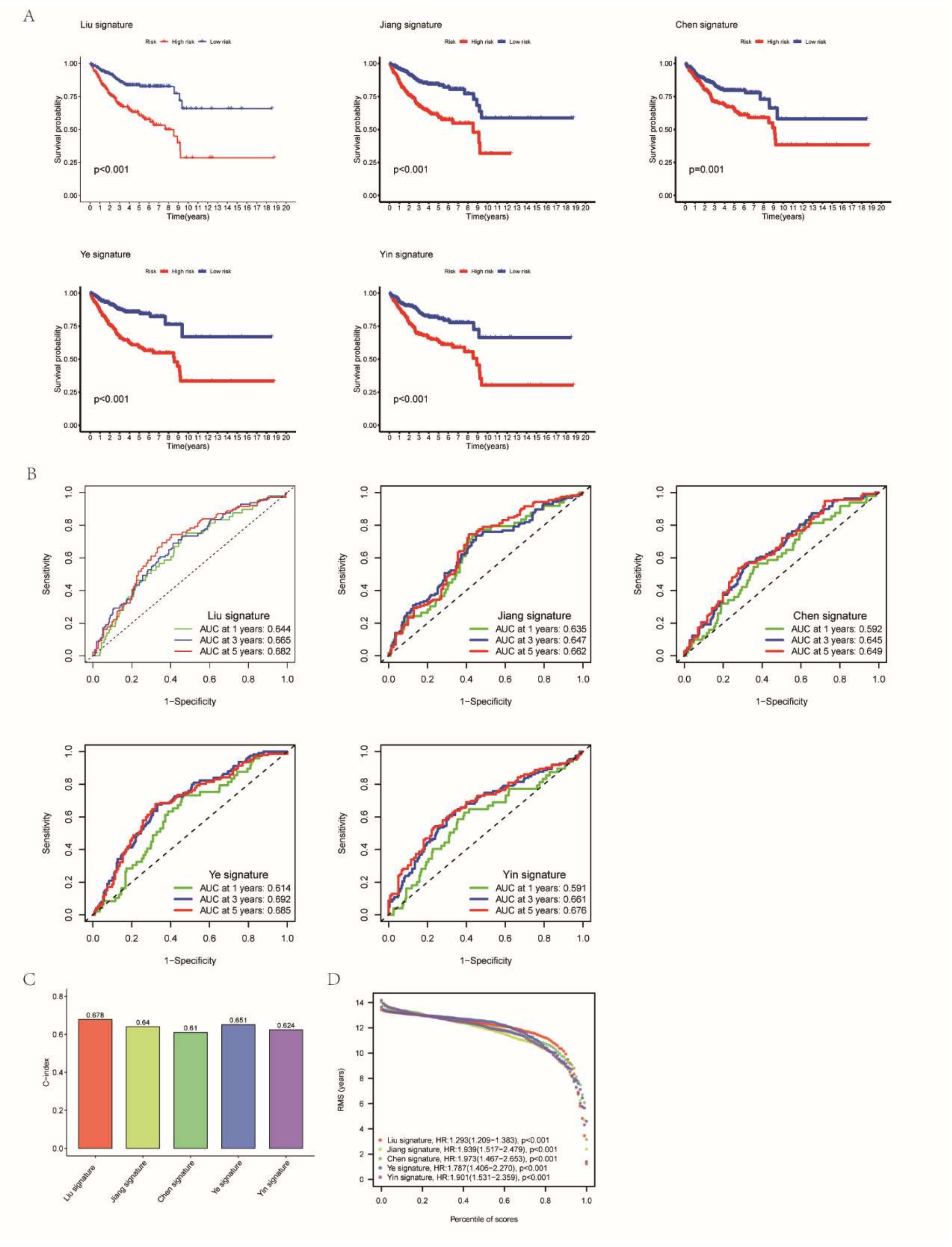

Figure S15 Comparison of RBS with other established models. K-M curves (A) and ROC curves (B) of EC predict models. (C) Concordance

index of the prognostic models. (D) Restricted mean survival time curve of the risk models.

Table S1 Univariate and multivariate Cox regression analyses of the prognosis-related factors in all set.

Variable	Univariable model				Multivariable model			
	HR	HR.95L	HR.95H	P-value	HR	HR.95L	HR.95H	*P*-value
age	1.7782	1.1121	2.8432	0.0162	1.4834	0.9116	2.4138	0.1124
histological_type	3.0435	2.0032	4.6242	0.0000	1.7483	1.1057	2.7643	0.0168
grade	3.3631	1.4671	7.7097	0.0042	1.3267	0.5447	3.2315	0.5337
stage	4.1162	2.7000	6.2754	0.0000	2.9852	1.8750	4.7528	0.0000
riskScore	1.3322	1.2371	1.4346	0.0000	1.2719	1.1710	1.3814	0.0000
